# Use of hypertonic glucose (10%) in the prevention of postoperative adhesions in rats

**DOI:** 10.1590/ACB360504

**Published:** 2021-06-25

**Authors:** João Nogueira, Alexandra de Oliveira do Carmo, Laura Sales Carvalho Lima, Lyvia Maria Rodrigues de Sousa Gomes, Ed Carlos Rey Moura, Caio Marcio Barros de Oliveira, Thiers Soares Raymundo, George Castro Figueira de Melo, Plinio da Cunha Leal

**Affiliations:** 1 PhD. Universidade Federal do Maranhão – College of Medicine – Department of Medicine I – São Luís (MA), Brazil.; 2 PhD. Universidade Federal do Maranhão – College of Medicine – Department of Medicine I – São Luís (MA), Brazil.; 3 PhD. Universidade Federal do Maranhão – College of Medicine – Department of Medicine I – São Luís (MA), Brazil.; 4 PhD. Universidade Federal do Maranhão – College of Medicine – Department of Medicine II – São Luís (MA), Brazil.; 5 PhD. Universidade Federal do Maranhão – College of Medicine – Department of Medicine I – São Luís (MA), Brazil.; 6 MD. Universidade Federal do Maranhão – College of Medicine – Department of Medicine III – São Luís (MA), Brazil.; 7 PhD. Universidade CEUMA – Department of Pathology –São Luís (MA), Brazil.

**Keywords:** Tissue Adhesions, Glucose Solution, Hypertonic, Dexamethasone

## Abstract

**Purpose:**

To evaluate the efficacy of hypertonic glucose (10%), alone or in combination with the corticoid dexamethasone, to prevent peritoneal adhesion following hysterectomy in rats.

**Methods:**

Forty-two adult rats underwent hysterectomy with peritoneal lavage: G1 – glucose (10%); G2 – glucose (10%) and dexamethasone 3 mg·kg^–1^; and G3 – physiological saline (PS) 0.9%.

**Results:**

In the macroscopic analysis after 14 days, G1 had a median score of 1, G2 of 1, and G3 of 2.5 (p < 0.0001), G3 compared to G1 and G2. There was no difference between groups after 28 days. In the microscopic analysis, the median vascular proliferation after 14 days was 2 for G1, 1 for G2, and 3 for G3 (p = 0.0037, G3 vs. G1 and G2). After 28 days, G1 showed a median vascular proliferation score of 2, G2 of 2.5, and G3 of 3 (p < 0.0001, G3 vs. G1 and G2). Regarding the inflammatory reaction after 14 days, G1 had a median score of 2, G2 of 1, and G3 of 3 (p = 0.7916). After 28 days, G1 had a median score of 0.5 (0–1.75), G2 of 1.5, and G3 of 2.5 (p < 0.0001, G3 vs. the others and G2 vs. G1). In the evaluation of fibrosis after 14 days, G1 had a median score of 1, G2 of 1, and G3 of 2.5 (p < 0.0001, G3 vs. G1and G2). After 28 days, G1 had a median fibrosis score of 1, G2: 2, and G3: 2.5 (p < 0.0001), G3 vs. the others andG2 vs. G1).

**Conclusions:**

The use of hypertonic glucose (10%) solution seems to reduce macroscopic and microscopic pelvic adhesions.

## Introduction

Surgical adhesions are bands of scar tissue that form between the surfaces of organs and are one of the main causes of postoperative complications^1^. Their pathological mechanism remains undefined, and preventive agents in clinical trials have failed to achieve effectiveness^1^,^2^.

Peritoneal adhesions are consequence of peritoneal irritation by crushing, thermic injury, foreign body implants, and other surgical or trauma, and may be considered as the pathological part of healing, following any peritoneal injury, particularly due to abdominal surgery^2^. In patients submitted to gynaecological surgery, 64% were readmitted within 10 years for a problem potentially related to adhesions and 2.9% of patients were readmitted for problems directly related to adhesions^1^,^2^. Besides, in patients that need emergency surgery and repetitive operations, severe adhesions can already be seen after the initial operation, leading to pain, infertility and bowel obstruction, limiting further exploration of the abdomen^1^,^2^.

In the process of normal peritoneal healing, several important steps occur, including fibrinolysis, proteolysis, and tissue remodelling^3^. Adhesions form mainly from abnormal repair of the peritoneum following repeated peritoneal lesions^3^. They may result in mesothelial defects or even increased vascular permeability, thus producing inflammatory exudate^3^. This exudate results in the presence of a mass of fibrin in the peritoneal cavity^2^. This mass is completely eliminated when the peritoneal fibrinolytic activity is normal^2^,^3^. Ischaemia or plasminogen activator inhibitor 1 and 2 overexpression, induced by inflammation, is the main cause of incomplete removal of the fibrin mass from the peritoneal cavity. Thus, when fibrin persists in the peritoneal cavity, fibroblasts proliferate in fibrin bands, which are organized into adhesions^3^,^4^.

Methods for the prevention of adhesions are divided into surgical techniques, physical barriers, and pharmacological therapies^2^. No pharmacological therapy has been approved for clinical use, which justifies further research into these methods^2^,^5^. Among pharmacological therapies, fibrinolytic, anticoagulant, and anti-inflammatory agents (corticosteroids and nonsteroidal anti-inflammatory agents) have been used, which theoretically have the potential to be auxiliary agents against adhesion formation^5^,^6^. Corticosteroids alter the inflammatory response, thereby reducing vascular permeability and consequently decreasing the secretion of cytokines and chemotactic factors^6^. They have been used alone or in combinations and are administered intraperitoneally or systemically^7^. They have also been used in postoperative lavage through the fallopian tubes and are effective in many but not in all experimental models^9^. However, evidence for substances that are effective in preventing adhesions in peritoneal, pelvic, or abdominal surgeries is still limited^6^–^8^.

Other pharmacological agents have drawn less attention and might deserve further study^9^,^10^. Hypertonic glucose has fibrinolytic action *in vitro*, so it could have value if used intraperitoneally to facilitate abdominal drainage in dialysis patients^10^,^11^. Animal experiments to evaluate the value of hypertonic glucose in the prevention of adhesions have been inconclusive^12^. Experimental studies have not uniformly demonstrated the satisfactory fibrinolytic action of the hypertonic glucose solution, and the results on the prevention of adhesions are conflicting^11^. In 1999, hypertonic glucose was shown to stimulate the synthesis of tissue plasminogen activator (t-PA) by human mesothelial cells in culture^12^. A few studies have confirmed this effect *in vivo* 9–12. One of the experimental studies performed indicated that this solution promotes faster, more resistant healing and less inflammatory reaction than 0.9% saline solution^13^. The mechanism of action remains controversial, but the low cost of the product, its large supply, and its easy clinical applicability contribute to its clinical use.

In the present study, the aim was to evaluate the efficacy of hypertonic glucose (10%), alone or in combination with the corticoid dexamethasone to prevent peritoneal adhesion following hysterectomy in rats, and to observe the degree of adhesion formation macroscopically and microscopically by evaluating the fibrosis process and vascular and inflammatory proliferation as a function of postoperative time.

## Methods

This was a prospective, blind, and analytical experimental study conducted at the Laboratory of Experimental Surgery of the University Hospital, Universidade Federal do Maranhão (UFMA) – Maternal and Child Unit. The project was approved by the Animal Ethics Committee (CEUA) of UFMA under protocol number 23115.011061/2018-48.

Animal rights were valued in the study according to the legal standards, specifically Law No. 11,794 of October 8, 2008, regulated on item VII, of the 1st, art. 225 of the Brazilian Federal Constitution, establishing procedures for the use of animals for teaching and/or scientific research purposes.

A total of 42 adult Wistar rats (3 months of age; females; virgins) from the Central Vivarium of UFMA were studied. Throughout the experiment, the animals were kept in the Laboratory of Experimental Surgery in acrylic cages measuring 30 × 30 × 17 cm^3^ with a maximum of six animals to a cage, where they were observed for 24 h under similar environmental conditions. The room had a 12-h light/dark cycle, and they were fed standard Purina feed for rodents and water *ad libitum*. The experiments were performed according to the Guide for the Care and Use of Laboratory Animals of the National Research Academy of the State of Washington, United States of America^14^.

The animals were randomly divided into three groups of 14, and all underwent hysterectomies. At the end of the surgery in the first group (G1), 10 mL of a hypertonic glucose (10%) solution was left in the pelvic cavity. The second group (G2) was given 10 mL of a 1:1 combination of hypertonic glucose (10%) and dexamethasone 3 mg·kg^–1^ (Decadron, Aché, São Paulo, SP, Brazil) according to Morris *et al* 15. The third group (G3), which were the controls, were given 10 mL of 0.9% saline solution.

The hysterectomies were performed with a ventral midline incision through the skin and peritoneum^16^. The sham surgery group received skin and peritoneum incisions only, each uterine horn was ligated with Nylon 4.0 (mononylon, black monofilament nylon, J&J Ethicon, São Paulo, SP, Brazil) and cut below the ovary and oviduct^16^. The uterus was then separated from the adjacent fat, and the uterocervical junction was ligated and cut above the cervix, at the base of the uterine body, after muscle incisions were sutured with dissolvable Vicryl 4.0 (polyglactin 910, J&J Ethicon, São Paulo, SP, Brazil) suture, and bupivacaine (Marcaine; Pfizer Pharmaceutical, São Paulo, SP, Brazil) was applied to the muscle incision prior to skin closure for all subjects^16^. The skin incision was closed with Nylon 4.0 (mononylon, black monofilament nylon, J&J Ethicon, São Paulo, SP, Brazil)^16^.

Before surgery, the animals were properly anaesthetized with the standard dose of a combination of 40 mg·kg^–1^ ketamine hydrochloride and 5 mg·kg^–1^ xylazine hydrochloride, given intramuscularly with a 13 × 4.5-mm hypodermic needle (Becton Dickinson, Paraná, Brazil) at the posterior border of the right lower limb of the animal^17^. The effectiveness of anaesthesia was confirmed by the loss of the corneal reflex and the tail reflex. Then, the animals were immobilized on a 20 × 30 cm wooden board. Next, manual epilation of the caudal abdominal region was performed, and antisepsis was performed with an alcohol solution of chlorhexidine digluconate 0.5% (Chlorhexidine Riohex, Rioquímica S/A, São Paulo, SP, Brazil).

After surgery, the animals were given analgesia with 15 mg·kg^–1^ ibuprofen orally at 24-h intervals. The animals were then randomly redistributed into two groups. In the first group, a new laparotomy was performed to analyse pelvic adhesions on the 14th postoperative day, while in the second group this was done on the 28th day. After this procedure, the rats were sacrificed with sodium thiopental (Tiopental) and evaluated. Euthanasia was performed according to resolution No. 714 of June 20, 2002, of the Federal Council of Veterinary Medicine of Brazil. Death was characterized by respiratory arrest and the complete absence of reflexes^17^. The carcasses were sent to incineration along with hospital waste destined for this purpose, according to the routine of the Presidente Dutra University Hospital, UFMA.

For the macroscopic analysis of the degree of adhesions, the Nair *et al* 18 classification was used, which gives them scores ranging from 0 to IV. Grade 0: complete absence of adhesions; grade I: one adhesion between two organs or between an organ and the abdominal wall; grade II: two adhesions between organs or between the organ and the abdominal wall; grade III: more than two adhesions between organs with each other or with the abdominal wall or a mass of generalized adhesions of the intestine without adhering to the abdominal wall; and grade IV: generalized adhesions between organs and the abdominal wall^18^.

Microscopic analysis was performed after surgical excision. For this purpose, the areas of adhesions were resected, or, when they were not visible, the areas where they should have formed were resected. These tissues were placed in containers with 10% buffered formalin. The tissue of all animals was sent for histopathological analysis by a single professional trained in the Pathology Service of the São Domingos do Maranhão Hospital. The pathologist was not aware of the group each piece belonged to, making it a blind evaluation.

The histological study was performed at the Pathology Laboratory, with the pieces processed in paraffin, cut to 3 μm and stained with haematoxylin–eosin (HE) and Masson’s trichrome (TM). In the histological evaluation, the parameters evaluated were fibrosis, inflammatory reaction, and vascular proliferation, assessed using a semiquantitative scale ranging from 0 to 3^19^,^20^.

The Kruskal–Wallis test was used to compare the groups, followed by Dunn’s post hoc test.

Throughout the study, the research team aimed to cause minimal physical and mental suffering to the animals.

## Results

### Macroscopic analysis

When comparing the presence of adhesions in the macroscopic analysis after 14 days, G1 had a median score of 1 (1–1), G2 had a median of 1 (1–2), and G3 had a median of 2.5 (2–3.75) (p < 0.0001, G3 vs. G1 and G2). There was no difference between G1 and G2.

**Figure 1:**
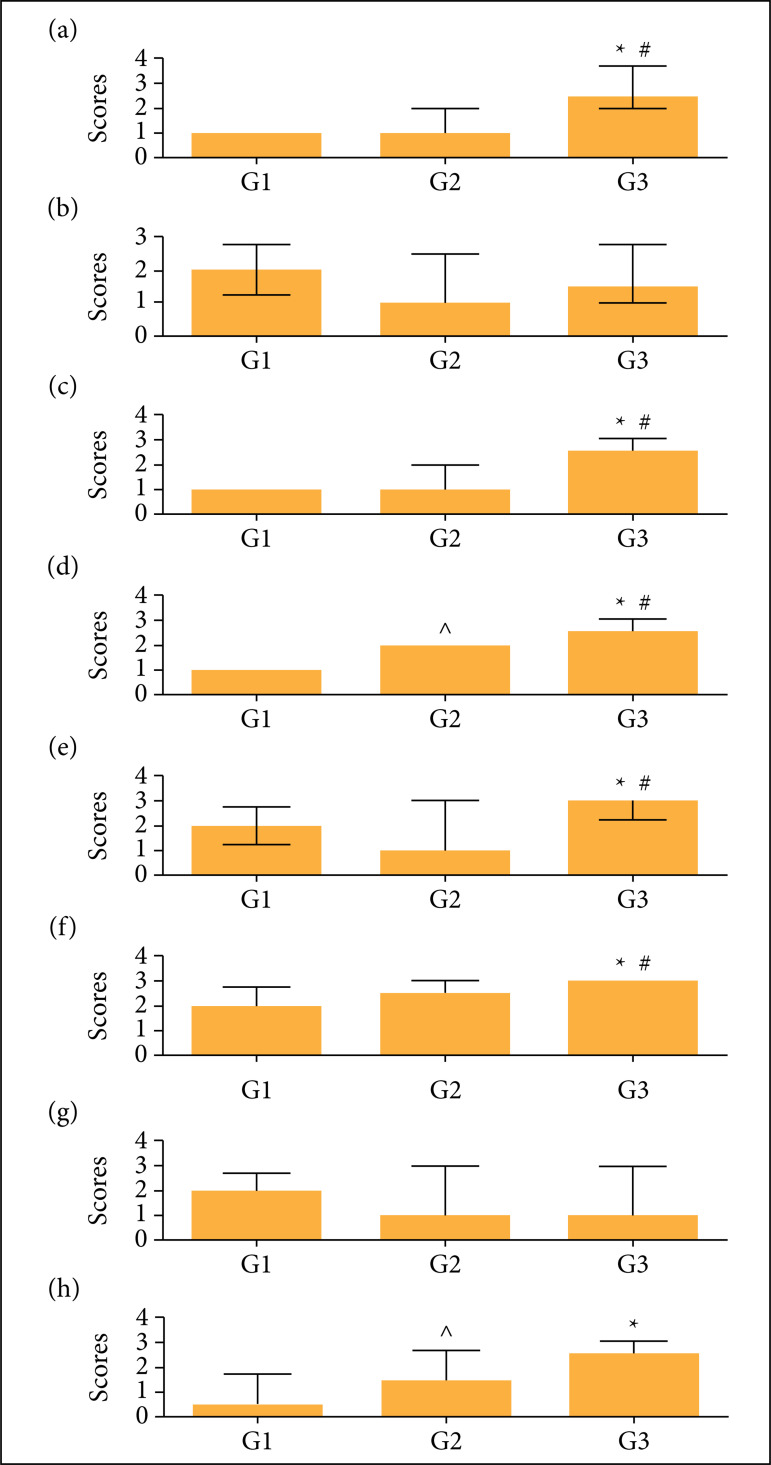
Comparison between groups. (a) Adhesions 14 days after surgery; (b) Adhesions 28 days after surgery; (c) Fibrosis 14 days after surgery; (d) Fibrosis 28 days after surgery; (e) Vascular proliferation 14 days after surgery; (f) Vascular proliferation 28 days after surgery; (g) Inflammatory reaction 14 days after surgery; (h) Inflammatory reaction 28 days after surgery. The Kruskal–Wallis test was used to compare the groups, followed by Dunn’s post hoc test. *Difference between G1 and G3; # difference between G2 and G3; ^ difference between G1 and G2.

**Figure 2:**
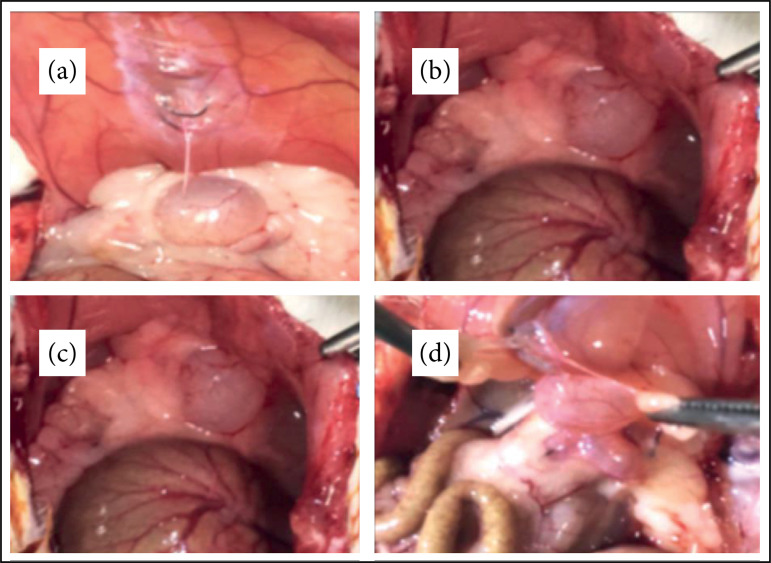
Image of adhesions of varying degrees according to the scale of Nair et al.^19^ **(a)** Group 1 – grade I;**(b)** Group 1 – grade II; **(c)** Group 2 – grade III; **(d)** Group 3 – grade IV. G1 – hypertonic glucose (10%) group, G2 – hypertonic glucose (10%) and dexamethasone group, G3 – 0.9% saline group.

When comparing the presence of adhesions in the macroscopic analysis after 28 days, G1 had a median score of 2 (1.25–2.75), G2 had a median of 1 (1–2.5), and G3 had a median of 1.5 (1–2.75) (p = 0.1411) (Figs. 1 and 2).

### Microscopic analysis

Regarding fibrosis, after 14 days, G1 had a median score of 1 (1–1), G2 had a median of 1 (1–2), and G3 had a median of 2.5 (2–3) (p < 0.0001, G3 vs. G1 and G2).

When comparing fibrosis after 28 days, G1 had a median score of 1 (1–1), G2 had a median of 2 (1.25–2), and G3 had a median of 2.5 (2–3) (p < 0.0001, G3 vs. the others and G2 vs. G1).

**Figure 3:**
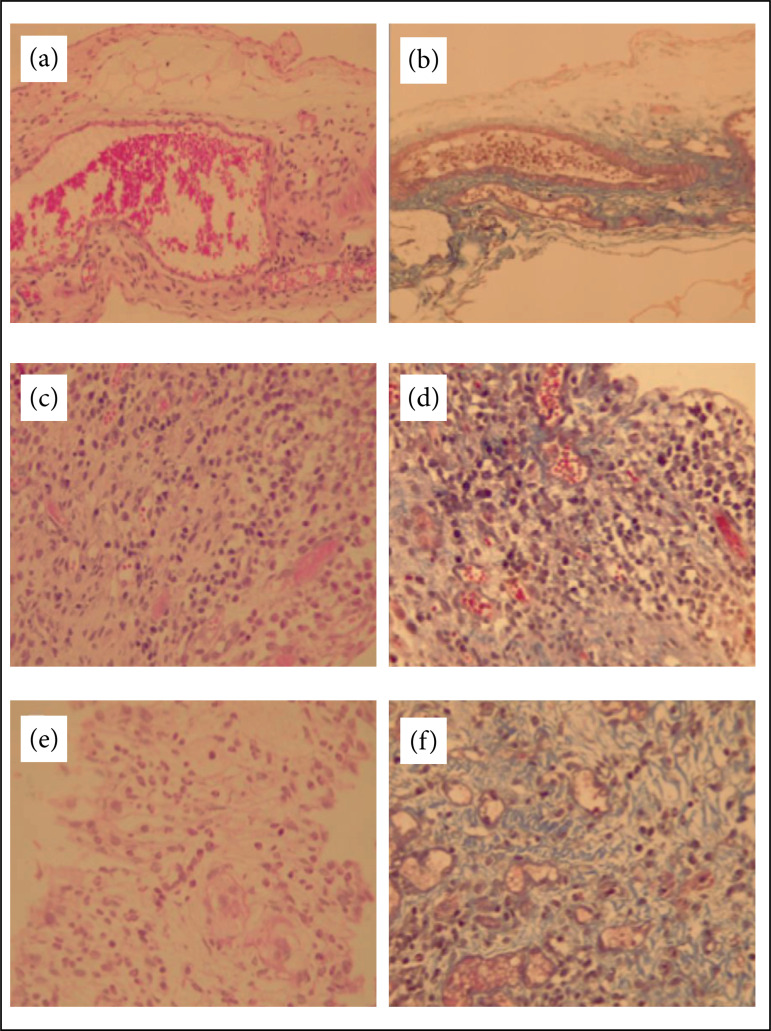
Photomicroscopy of pelvic adhesions in Wistar rats. (a) G1 showing an absence of significant inflammatory infiltrate and vascular congestion (HE-200×); (b) G1 showing an absence of significant fibrosis (TM-200×); (c) G2 showing moderate chronic inflammatory infiltrate with moderate angiogenesis (HE-400×); (d) G2 showing moderate angiogenesis and fibrosis (TM-400×); (e) G3 showing moderate mixed inflammatory infiltrate (HE-400×); (f) G3 with marked angiogenesis and moderate fibrosis (TM-400×). G1 – hypertonic glucose (10%) group, G2 – hypertonic glucose (10%) and dexamethasone group, G3 – 0.9% saline solution group. HE – haematoxylin eosin, MT – Masson’s trichrome.

Regarding vascular proliferation after 14 days, G1 had a median score of 2 (1.25–2.75), G2 had a median of 1 (1–3), and G3 had a median of 3 (2.25–3) (p = 0.0037, G3 vs. G1 and G2). There was no difference between G1 and G2 (Figs. 1 and 3).

When comparing vascular proliferation after 28 days, G1 had a median score of 2 (0.5–2.75), G2 had a median of 2.5 (2–3), and G3 had a median of 3 (3–3) (p < 0.0001, G3 vs. G1 and G2) (Fig. 1).

The inflammatory reaction after 14 days was similar between groups: G1 had a median score of 2 (1–2.75), G2 had a median of 1 (1–3), and G3 had a median of 3 (1–3) (p = 0.7916) (Figs. 1 and 3).

When comparing the inflammatory reaction after 28 days, G1 had a median score of 0.5 (0–1.75), G2 had a median of 1.5 (1–2.75), and G3 had a median of 2.5 (2–3) (p < 0.0001, G3 vs. the others and G2 vs. G1) (Figs. 1 and 3).

## Discussion

The use of hypertonic glucose (10%) alone (G1) or combined with dexamethasone (G2) macroscopically reduced adhesions on day 14 postoperatively in Wistar rats subjected to hysterectomy. On microscopy, G1 and G2 showed less vascular proliferation and fibrosis on the 14th and 28th postoperative days than the control rats (G3) and a minor inflammatory process on the 28th postoperative day. In addition, it should be noted that there was less fibrosis and inflammation on the 28th postoperative day in G1 than in G2.

Postoperative adhesion formation is still a major challenge for the healthcare setting and has consequences for patients, surgeons, and the health system^1^,^2^. Attempts to intervene in this process, as a means of prevention, require a deep understanding of its pathophysiology, the surgical techniques used, and the nature of the materials and substances used for prevention^21^. Although procedures and technologies have been improved to reduce the formation of adhesions, such as laparoscopic or robotic surgery, minimally invasive surgery is not always applicable or available^21^. Any prevention strategy should be safe, effective, practical, and economical. Among the preventive methods, physical barriers, mainly through the use of solutions, are the most studied, widespread, and currently used method^2^.

The physical barrier methods available on the market are divided into solids and liquids. The solids can be absorbable (carboxymethylcellulose and oxidized regenerated cellulose) or nonabsorbable (expanded polytetrafluoroethylene)^2^. The liquid barriers are glycol polyethylene and solutions of icodextrin, hyaluronic acid, and plant polysaccharides^2^,^5^. These materials increase the cost of surgery and have unreliable effects in the prevention of adhesions^2^,^6^. Several other substances with different mechanisms of action, such as cow peritoneum, amniotic membranes or liquids, oxidized celluloses, olive oil, soybean oil, starch, glycerol, honey, diluted glucose, corticosteroids, and many others have been tried to prevent the formation of postoperative peritoneal adhesion^2^,^6^–^8^,^12^,^13^,^22^,^25^.

Ischemia or plasminogen activator inhibitor 1 and 2 overexpression, induced by inflammation, is the main cause of incomplete elimination of the fibrin mass from the peritoneal cavity^3^,^2^. Thus, when fibrin persists in the peritoneal cavity, fibroblasts proliferate in fibrin bands, which are organized into adhesions^3^–^4^.

Sitter *et al* 12, evaluating the physical and chemical irritation of the peritoneum using glucose-based hyperosmolar dialysis solutions, observed nonbacterial serositis with fibrinous exudate and found that human peritoneal mesothelial cells (HPMCs) play an important role in maintaining the balance between peritoneal generation and fibrin degradation by expressing the fibrinolytic enzyme tissue plasminogen activator (t-PA), as well as plasminogen activator inhibitor-1 (PAI-1). These authors analysed the effect of D-glucose and metabolically inert monosaccharides in the synthesis of t-PA and PAI-1 in cultured HPMCs. They concluded that hyperosmolarity induces t-PA (but not PAI-1) in HPMCs through a regulatory mechanism that requires active protein kinase C (PKC), but whose main pathway does not involve the mitogen-activated protein kinase (MAPK) cascade. Based on this premise, some studies have evaluated the use of hypertonic glucose in the prevention of pelvic adhesions, but they are few and inconclusive^9^–^12^.

Experimental studies evaluating the macroscopic and microscopic changes in the mesentery and parietal peritoneum in rats have administered 10 and 25% hypertonic glucose aqueous solutions to the peritoneal cavity of rats and have observed the absence of tissue necrosis and inflammation with the same intensity in the mesentery and parietal peritoneum^13^. This result led to the choice of only one dose of hypertonic glucose (10%) in this study.

Corticosteroids, when administered alone or in combinations intraperitoneally, have been investigated in experimental and clinical studies^8^,^23^. They have had questionable efficacy associated with immunosuppression and delayed wound healing^8^,^23^. The controlled release of dexamethasone reduced, but did not eliminate adhesion formation in an experimental study^23^. Other evidence still suggests that steroids can decrease abdominal adhesions after surgery^8^. In practice, corticosteroids are not often used to prevent adhesions, as the literature leaves doubts about their function and their efficacy. Due to the lack of firm conclusions about corticosteroids in the prevention of adhesions, this study included a group that received dexamethasone and hypertonic glucose (10%) (G2) to see if this combination would decrease adhesions.

There was an increase in the degree of adhesions in G3 compared to G1 and G2 on the 14th postoperative day, but there was no difference on the 28th day. Thus, hypertonic glucose (10%) alone or in combination with dexamethasone was shown to reduce adhesions in the immediate postoperative period, although this effect did not last until the late postoperative period (Fig. 1).

The glucose 10% solution alone or in combination with dexamethasone led to a lower degree of fibrosis than the control (saline) on both the 14th and 28th postoperative days. In addition, the hypertonic glucose (10%) group (G1) showed less fibrosis than the group combining glucose 10% with dexamethasone (G2), which is in agreement with the finding that corticosteroids increase the risk of fibrosis^2^,^8^,^23^

Regarding vascular proliferation, there was greater proliferation in G3 than in G1 and G2 on both the 14th and 28th postoperative days. This finding contrasts with some studies that suggest that hyperglycaemia increases angiogenesis and that glucose increases angiogenesis and accelerates healing in normal and diabetic rats^24^–^27^.

In addition, the inflammatory process was greater in G3 than in G1 and G2 and was greater in G2 than in G1 on the 28th postoperative day. This result in the hypertonic glucose-only group was unexpected because, according to the medical literature, glucose promotes increased inflammatory processes^8^,^26^,^27^.

This study had some limitations. First, evaluation after six weeks would be more appropriate. Second, the evaluation of the adhesions, fibrosis, vascularization and inflammation, which in the majority of the researches vary from 3 to 5, and a score of 3 was chosen in the present study. Besides, other biomarkers could show more insights.

## Conclusion

It is possible to conclude that, in this experimental study, hypertonic glucose (10%) solution seemed to reduce macroscopic and microscopic pelvic adhesions when compared to saline solution, while the combination of hypertonic glucose (10%) with dexamethasone did not show the expected enhancing effect.
